# Direct-acting antiviral treatment impact on glycemic control in HCV-infected T2DM patients: An observational study with Mendelian randomization analysis

**DOI:** 10.1097/MD.0000000000044319

**Published:** 2025-10-31

**Authors:** Jie Tang, Jian Zhou, Liyao Zhu, Yan Zhao, Guoping Yin, Rui Zhang

**Affiliations:** a Department of Hepatology, The Fourth Hospital of Huai’an, Huai’an, Jiangsu, P. R. China; b Department of Gastroenterology, Huai’an Hospital of Huai’an City, Huai’an, Jiangsu, P. R. China; c Department of Anesthesiology, Nanjing Second Hospital, Nanjing, Jiangsu, P. R. China.

**Keywords:** direct-acting antivirals, glycemic control, hepatitis C virus, retrospective study, type 2 diabetes mellitus

## Abstract

Hepatitis C virus (HCV) infection is a major global health issue, associated with various metabolic disorders, including type 2 diabetes mellitus (T2DM). This study explores the impact of direct-acting antiviral agents (DAAs) on diabetes management in patients coinfected with HCV and T2DM. This study comprised a cross-sectional analysis of NHANES database (1999–2018) participants with HCV antibody-positive status and a retrospective cohort of T2DM patients achieving sustained virological response after DAAs treatment at The Fourth Hospital of Huai’an (2020–2023). We conducted Mendelian randomization analysis using expression Quantitative Trait Loci data (eQTLGen, GTEx) and protein quantitative trait loci data (Iceland Protein, UK Biobank) of DAAs target genes from blood and liver samples. The cross-sectional NHANES analysis assessed antiviral therapy effects on diabetes indicators, while the retrospective study evaluated changes in hemoglobin A1c (HbA1c) and fasting glucose at 6, 12, and 18 months post-DAA treatment. Mendelian randomization highlighted the potential role of the CXCL10 gene in modulating HbA1c levels. The cross-sectional NHANES study found no significant associations between interferon/ribavirin use and diabetes indicators. In the retrospective study, significant short-term improvements in HbA1c and fasting glucose were observed within 6 months of DAA therapy; however, these were not sustained over 12 to 18 months. Notably, patients with cirrhosis or abnormal BMI showed no short-term metabolic improvements. The study underscores the transient nature of the metabolic improvements following HCV eradication in T2DM patients and stresses the importance of comprehensive management strategies to sustain glycemic control in the long term.

## 1. Introduction

Hepatitis C virus (HCV) infection remains a significant global public health concern, affecting millions of individuals. The association between HCV infection and various metabolic disorders, notably type 2 diabetes mellitus (T2DM), has been extensively documented, adding to the disease burden and complexity of management for affected individuals.^[1]^ Over the years, the link between T2DM and HCV infection has garnered widespread attention, with research indicating a higher risk of developing T2DM among HCV-infected individuals compared to the general population.^[2]^ Although the exact mechanisms remain incompletely understood, HCV infection is currently recognized to increase the risk of T2DM through effects on pancreatic β-cell function or indirectly through systemic inflammation and insulin resistance.^[3]^

The metabolic impact of HCV infection is profound and multifaceted. HCV adversely affects host metabolic function through pancreatic β-cell destruction, oxidative stress induction, inflammatory response triggering, and disruption of normal lipid metabolism.^[3–6]^ Notably, liver dysfunction caused by HCV infection further exacerbates metabolic disorders, creating a vicious cycle. As the core organ of glucose metabolism, liver dysfunction significantly affects insulin sensitivity and glucose homeostasis. Liver fibrosis and cirrhosis induced by chronic HCV infection not only reduce the liver’s responsiveness to insulin but also accelerate the progression of diabetes through multiple mechanisms, including altered glycogen synthesis and storage, gluconeogenesis regulation, and insulin degradation.^[7]^

Over the past decades, antiviral treatment for HCV has made significant progress, evolving from traditional interferon and ribavirin regimens to modern direct-acting antivirals (DAAs).^[8]^ DAAs therapy has become the preferred method for treating HCV infection due to its high efficacy and better tolerability. Although DAAs therapy can effectively clear viral load and mitigate these adverse effects, insulin resistance and β-cell dysfunction caused by chronic HCV infection may not fully recover, particularly in patients with severe metabolic disorders or significant liver dysfunction.^[9]^

Current reports on the impact of these treatments on glycemic control in patients with concomitant T2DM are conflicting. Some studies suggested that successful antiviral treatment can improve glycemic control and diabetes status, while others have not found such an association.^[10–12]^ This study aims to comprehensively explore whether antiviral therapy affects HCV-infected patients with concomitant T2DM from pharmacogenomic and epidemiological perspectives, and to investigate the durability of this improvement, through multiple databases and statistical models. We hope to provide deeper insights and evidence for the comprehensive treatment and management of patients with HCV and concomitant T2DM.

## 2. Methods

### 2.1. Mendelian randomization analysis

We initially predicted the drug targets of interferon, ribavirin, and DAAs through the DGIdb database.^[13]^ Subsequently, we obtained the expression Quantitative Trait Loci (eQTL) data of these genes in blood samples from the eQTLGen Consortium,^[14]^ and eQTL data in blood or liver samples from GTEx Portal.^[15]^ Additionally, we obtained protein Quantitative Trait Loci data of these genes from the Iceland Protein Database^[16]^ and UK Biobank database.^[17]^ Outcome variables were derived from GWAS data on diabetes, fasting glucose, and hemoglobin A1c (HbA1c) obtained from the UK Biobank catalogue.^[17]^ We utilized eQTL or pQTL as instruments and applied the summary data-based Mendelian randomization (SMR) method to generate effect estimates,^[18]^ exploring the relationship between gene expression levels and outcome variables. Allele coordination and analysis were conducted using the SMR software (version 1.31; University of Queensland, Brisbane, Queensland, Australia).^[19]^ The heterogeneity in dependent instruments (HEIDI) test was performed to examine whether the observed association between gene expression and outcomes was due to linkage. A HEIDI test with *P* < .01 indicated that the association might be due to linkage.^[20]^

### 2.2. Design and statistics of cross-sectional study

The cross-sectional study aimed to provide a representative sample of the U.S. civilian population. We utilized data from the 1999 to 2018 NHANES cycles, including demographic details, self-reported antiviral drug use, and diabetes indicators.^[21]^ This study received approval from the Ethics Committee of the Centers for Disease Control and Prevention, with participants providing informed consent. Our analysis focused on individuals positive for HCV antibodies, with additional indicators including interferon or ribavirin use, HbA1c, fasting glucose, T2DM, and confounding factors such as age, gender, race, education, smoking, alcohol, and hypertension. Detection of HbA1c and fasting glucose was performed using serum samples collected in the morning, frozen at −30 °C, and then transported to the National Center for Environmental Health for analysis. The analysis involved fasting serum biochemistry profiles measured using the timed endpoint method on the DxC800. Hypertension was defined by the following criteria: 3 examinations, a physician diagnosis, or blood pressure > 130/80 mm Hg with antihypertensive medication use. Diabetes was defined as fasting blood glucose > 126 mg/dL, glycosylated hemoglobin > 6.5%, physician diagnosis, or antidiabetic medication use. Analysis was conducted using R 4.2.3 (version 4.2.3; R Foundation for Statistical Computing, Vienna, Austria) and EmpowerStats 4.0 (version 4.0; X&Y Solutions, Inc., Boston). Among 37 eligible participants, we investigated the potential association between antiviral drug use in HCV patients and T2D-related indicators after adjusting and unadjusting for confounding factors using Mann–Whitney *U* test and general linear regression (SURVEYREG procedure).

### 2.3. Retrospective clinical study

#### 2.3.1. Study population and design

A retrospective study was conducted on T2D patients with HCV infection who underwent DAA treatment at the Huai’an Infectious Disease Hospital between January 1, 2020, and October 30, 2023. Electronic medical record searches identified all patients receiving DAA treatment with Epclusa (Sofosbuvir/Vepatasvir), excluding those with type 1 diabetes mellitus (T1DM) and patients lacking key data in electronic medical records (i.e., no pre- or posttreatment HbA1c or fasting glucose values). Patients who did not achieve sustained virological response (SVR) at 12 weeks after treatment were considered treatment failures and were also excluded. The medical records of each eligible patient were reviewed, and demographic characteristics including age, gender, BMI, cirrhosis, HCV genotype, pretreatment HCV RNA (IU/mL), as well as albumin (g/L), aspartate aminotransferase (AST) (U/L), alanine aminotransferase (ALT) (U/L), total cholesterol (mmol/L), triglycerides (mmol/L), high-density lipoprotein cholesterol (mmol/L), low-density lipoprotein (LDL) cholesterol (mmol/L), creatinine (µmol/L), insulin (pmol/L), and total bilirubin (µmol/L) before and after treatment were recorded. We calculated insulin resistance indicators including homeostatic model assessment of insulin resistance (HOMA-IR) and Quantitative Insulin Sensitivity Check Index (QUICKI), and recorded HbA1c (%) levels and diabetes medication dosages at baseline, 6, 12, and 18 months after treatment initiation. HOMA-IR and QUICKI were calculated using the following formulas^[22,23]^: HOMA-IR = fasting insulin (µIU/mL) × fasting glucose (mmol/L)/22.5, and QUICKI = 1/(log [fasting insulin [µIU/mL]] + log [fasting glucose [mg/dL]]).

Renal dysfunction was diagnosed according to the KDIGO (Kidney Disease: Improving Global Outcomes) guidelines, defined as serum creatinine levels > 133 µmol/L in males or >124 µmol/L in females.^[24]^ Dyslipidemia was diagnosed based on NCEP-ATP III criteria, meeting any of the following conditions: triglycerides ≥ 1.69 mmol/L; total cholesterol ≥ 5.17 mmol/L; LDL cholesterol ≥ 3.37 mmol/L; or decreased high-density lipoprotein cholesterol (<1.04 mmol/L in males, <1.29 mmol/L in females).^[25]^ The study protocol and experiments were approved by the Institutional and Local Ethics Committee of The Fourth Hospital of Huai’an (HASY2020002).

#### 2.3.2. Statistical methods

Categorical variables were compared using Chi-square test. For continuous variables, Student *t* test was used when data followed normal distribution; when data were not normally distributed, Wilcoxon signed-rank test was applied for paired samples, and Mann–Whitney *U* test was used for independent samples.^[26]^ We initially compared the most recent (6-month) HbA1c and fasting glucose levels before and during treatment period, followed by assessing the persistence of response at 12 and 18 months. Furthermore, the strength of the effect of different posttreatment periods on HbA1c and fasting glucose levels was considered using generalized linear regression, while incorporating demographic-related factors to assess whether the results were influenced by covariates. We compared baseline characteristics between patients with and without glycemic improvement after DAAs treatment. Demographic factors were incorporated to evaluate the potential influence of covariates on treatment outcomes. Stratified analyses were performed to assess differences in hyperglycemia and HbA1c levels between baseline and 6 months posttreatment across different clinical categories (BMI, cirrhosis status, AST, and ALT). Improved diabetes control was defined as a reduction in HbA1c > 0.5%, or changes in diabetes treatment (reduced dose or number of diabetes medications, or switching from insulin to oral medications). Statistical significance was defined as *P* = .05.

## 3. Results

### 3.1. Mendelian randomization

In the DGIdb database, we identified 15, 37, and 28 potential target genes for interferon, ribavirin, and DAAs, respectively. After eliminating duplicates, a total of 73 unique genes were determined (Table S1, Supplemental Digital Content, https://links.lww.com/MD/Q377). Instrumental variables for these genes were sourced from blood or liver samples in the eQTLGen Consortium and GTEx databases for 41, 49, and 61 genes, and from the Iceland Protein Database and UK Biobank protein database for 42 and 31 genes, respectively.

**Figure 1.**
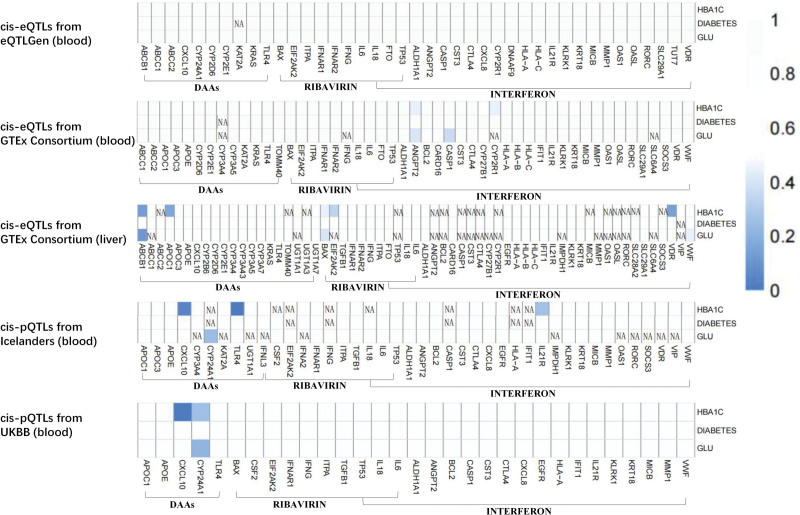
*P*-value heatmap of summary-data-based Mendelian randomization (SMR) association between anti-HCV medicine target genes and diabetes, HbA1c, glucose. HbA1c = hemoglobin A1c, HCV = hepatitis C virus.

SMR analysis revealed a statistically significant association between elevated protein expression of the CXCL10 gene in blood and reduced HbA1c levels in both the Iceland Protein Database and the UK Biobank database. Specifically, the effect sizes were β = −7.31 × 10^−3^, 95% CI: −1.43 × 10^−2^ to −2.72 × 10^−4^, *P* = 4.18 × 10^−2^, and β = −8.06 × 10^−3^, 95% CI: −1.58 × 10^−2^ to −3.16 × 10^−4^, *P* = 4.14 × 10^−2^ (Fig. 1). None of the other genes demonstrated significant causal relationships with HbA1c, fasting glucose, or T2DM. HEIDI results for the CXCL10 gene in both databases indicated *P*-values of 1.00 and .63, respectively, confirming that the significant associations observed were not attributable to linkage (*P* > .01) (Table S2, Supplemental Digital Content, https://links.lww.com/MD/Q378).

### 3.2. Cross-sectional study

A total of 37 NHANES participants were included in the screening process, with demographic, HCV medication data, and diabetes-related indicators documented. Among them, 26 were male and 11 were female. Twenty-one participants self-reported using interferon or ribavirin. Approximately half of the patients had a current diagnosis of diabetes, and 15 were considered hypertensive. The majority of participants were under 60 years old (26 out of 37). Thirty-two participants were classified as overweight or obese, while 19 reported never drinking alcohol or consuming alcohol less than once a month. Seventeen participants had a high school education, and most had an income greater than $20,000. Table S3, Supplemental Digital Content, https://links.lww.com/MD/Q377 provides detailed weight information.

Mann–Whitney *U* tests revealed no significant differences between interferon and ribavirin HCV users and nonusers regarding HbA1c, insulin, and fasting glucose levels. Similarly, linear regression models indicated no significant association between higher HbA1c, insulin, fasting glucose, and the use of interferon and ribavirin. After adjusting for covariates in the regression model (either diabetes alone or a combination of demographic factors, diabetes, hypertension, alcohol, and smoking), no significant associations were observed, although the confidence intervals showed a slight increase (Table 1).

**Table 1:** Mann–Whitney *U* test of fasting glucose, insulin, and HbA1c between participants with or without treatment of interferon or ribavirin, and generalized linear regression beta coefficients comparing fasting glucose, insulin, and HbA1c values between participants with and without interferon or ribavirin treatment.

	Treatment		Nontreatment		
Mann–Whitney U test					
	Mean (SD)	N	Mean (SD)	N	*P*-value
Fasting glucose (mmol/L)	112.31 (42.64)	21	110.16 (25.89)	16	.86
Insulin (pmol/L)	21.19 (13.88)	21	14.91 (13.55)	16	.18
HbA1c (%)	5.85 (1.07)	21	5.67 (0.87)	16	.59

Generalized linear regression					
	β (95% CI)	*P*-value	β (95% CI)	*P*-value	
*Non-adjustation*					
Fasting glucose (mmol/L)	−2.16 (−25.85, 21.53)	.86	−2.71 (−30.10, 24.67)	.85	
Insulin (μIU/mL)	−6.28 (−15.22, 2.66)	.18	−4.75 (−14.84, 5.34)	.36	
HbA1c (%)	−0.18 (−0.82, 0.46)	.86	−0.42 (−1.00, 0.15)	.16	
*Adjustation for diabetes*					
Fasting glucose (mmol/L)	0.69 (−20.69, 22.06)	.95	−2.73 (−26.79, 21.33)	.83	
Insulin (μIU/mL)	−5.57 (−14.24, 3.09)	.22	−4.75 (−14.56, 5.06)	.35	
HbA1c (%)	−0.11 (−0.70, 0.49)	.72	−0.42 (−0.94, 0.10)	.12	
*Adjustation for all confounding factors*					
Fasting glucose (mmol/L)	7.73 (−19.95, 35.40)	.59	2.91 (−21.76, 27.57)	.82	
Insulin (μIU/mL)	−8.54 (−17.55, 0.47)	.09	−9.88 (−18.85, −0.90)	.05	
HbA1c (%)	−0.04 (−0.95, 0.88)	.94	−0.18 (−1.07, 0.71)	.70	

CI = confidence interval, HbA1c = hemoglobin A1c, SD = standard deviation.

### 3.3. Retrospective study

During the years 2020 to 2023, we firstly identified 74 patients diagnosed with HCV combined with T2DM who were treated with DAAs treatment at The Fourth Hospital of Huai’an; 2 cases were excluded due to diagnosis of T1DM; 10 cases were excluded due to lack of pretreatment or posttreatment HbA1c records. Among the remaining 62 patients, 2 patients were excluded due to treatment failure, with an SVR occurrence rate of 60/62. Table 2 details the clinical characteristics of these patients. Half were under 60 years old, evenly distributed between genders, all of Han ethnicity, with the majority having genotype 1 HCV (70%) and 36.67% exhibiting cirrhosis. Overweight or obesity was present in 48.33%. Among the study participants, oral antidiabetic medications were used by all patients, while 40% additionally required insulin therapy. All 60 patients had data for fasting glucose and HbA1c at baseline and 6 months posttreatment. 46 patients had fasting glucose and HbA1c data at 12 months posttreatment, and 28 patients had data available at 18 months posttreatment. Hepatorenal biochemical parameters, and lipid profile data were available for 52 patients at both baseline and posttreatment. Insulin data were available for 26 patients before and after treatment.

**Table 2:** Demographic and clinical characteristics of diabetic patients successfully treated for hepatitis C virus with DAAs from 2020 to 2023.

Characteristic	All patients successfully treated for hepatitis C virus with direct-acting antivirals (n = 60)			
	*N*		Percent (%)	
*Age*				
60− yr	27		45.00%	
60+ yr	33		55.00%	
*Gender*				
Male	30		50.00%	
Female	30		50.00%	
*Hepatitis C virus genotype*				
1	42		70.00%	
2	14		23.33%	
3	2		3.33%	
4	1		1.67%	
6	1		1.67%	
*BMI*				
Underweight (<18.5)	1		1.67%	
Normal (18.5 to <24)	30		50.00%	
Overweight (24 to <30)	29		48.33%	
*Cirrhosis*				
Yes	22		36.67%	
No	38		63.33%	
*Prescribed any diabetes medication*				
Oral medications	60		100.00%	
Insulin injection	24		40.00%	

	Mean	SD	Median	Q1–Q3
Total bilirubin (µmol/L)	34.27	44.14	17.90	12.10–27.68
Albumin (g/L)	38.07	6.47	38.50	33.00–42.93
AST (U/L)	95.23	155.01	39.00	24.75–73.25
ALT (U/L)	110.75	146.35	54.00	28.00–97.75
Fasting glucose (mmol/L)	8.24	3.35	7.58	6.20–9.18
HBA1C (%)	7.53	1.69	7.20	6.30–7.930
Insulin (pmol/L)	72.49	41.44	69.60	42.84–87.98
HOMA-IR	3.74	2.24	3.35	2.00–4.76
QUICKI	0.32	0.02	0.32	0.30–0.34
Total cholesterol (mmol/L)	4.40	1.20	4.21	3.71–4.85
Triglycerides (mmol/L)	1.37	0.74	1.24	0.87–1.69
HDL cholesterol (mmol/L)	1.15	0.28	1.10	0.94–1.35
LDL cholesterol (mmol/L)	2.71	0.71	2.70	2.27–3.10
Creatinine (µmol/L)	119.91	33.49	124.80	103.13–140.05

ALT = alanine aminotransferase, AST = aspartate aminotransferase, BMI = body mass index, DAAs = direct-acting antiviral agents, HbA1c = hemoglobin A1c, HDL = high-density lipoprotein, HOMA-IR = homeostatic model assessment of insulin resistance, LDL = low-density lipoprotein, Q1–Q3 = first quartile-third quartile, QUICKI = Quantitative Insulin Sensitivity Check Index, SD = standard deviation.

**Figure 2.**
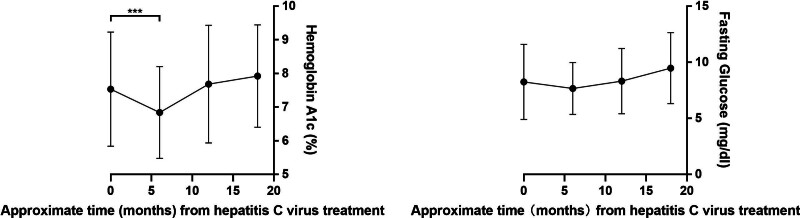
Mean fasting glucose and hemoglobin A1c value and 95% confidence interval before and after 6, 12, 18-month intervals of DAAs treatment. DAAs = direct-acting antiviral agents.

The initial mean fasting glucose level prior to treatment was 8.24 (mmol/L) ± 3.35, and the HbA1c was 7.53% ± 1.69 (Table 2). Fasting glucose showed no significant changes at 6 months posttreatment (8.24 [6.20–9.18] vs 7.20 [6.41–8.37], *P* = .14) and 12 months (8.24 [6.20–9.18] vs 7.81 [6.57–8.77], *P* = .83), but increased significantly at 18 months (8.24 [6.20–9.18] vs 8.65 [7.36–10.78], *P* = .04). HbA1c decreased significantly at 6 months (7.53 [6.30–7.93] vs 6.65 [6.05–7.43], *P* < .01), returned to baseline at 12 months (7.53 [6.30–7.93] vs 7.25 [6.60–8.00], *P* = .75), and increased significantly at 18 months (7.53 [6.30–7.93] vs 7.55 [6.75–8.55], *P* = .01). (Table 3). Figure 2 illustrates the temporal dynamics of HbA1c and fasting glucose, showing a significant reduction 6 months posttreatment; however, this significance was not maintained at 12 and 18 months. Generalized linear models accounting for repeated measures over time identified a significant reduction in HbA1c 6 months after initiating HCV treatment (−0.7 [−1.2, −0.1], *P* = .02), whereas changes in fasting glucose were not statistically significant (−0.56 [−1.60, 0.48], *P* = .29). Notably, there was a significant increase in HbA1c at 12 months relative to baseline (0.76 [0.09, 1.42], *P* = .03). No significant differences in HbA1c or fasting glucose were observed at other evaluation points compared to pretreatment values, as detailed in Table 3.

**Table 3:** Wilcoxon signed-rank test and generalized linear regression beta coefficients assessing fasting glucose and hemoglobin A1c value before and after 6, 12, 18-month intervals of DAAs treatment.

Wilcoxon signed-rank test			
	Median (Q1–Q3)		*P*-value
*Fasting glucose (mmol/L*)			
Pretreatment vs posttreatment (6 months)	8.24 (6.20–9.18)	7.20 (6.41, 8.37)	.14
Pretreatment vs posttreatment (12 months)	8.24 (6.20–9.18)	7.81 (6.57, 8.77)	.83
Pretreatment vs posttreatment (18 months)	8.24 (6.20–9.18)	8.65 (7.36, 10.78)	.04*
*HbA1c (%*)			
Pretreatment vs posttreatment (6 months)	7.53 (6.30–7.93)	6.65 (6.05, 7.43)	<.01*
Pretreatment vs posttreatment (12 months)	7.53 (6.30–7.93)	7.25 (6.60, 8.00)	.75
Pretreatment vs posttreatment (18 months)	7.53 (6.30–7.93)	7.55 (6.75, 8.55)	.01*

Generalized linear regression			
	Sample	β (95% CI)	*P*-value
*Fasting glucose (mmol/L*)			
Posttreatment (6 months) vs pretreatment	60	−0.56 (−1.60, 0.48)	.29
Posttreatment (12 months) vs pretreatment	46	0.33 (−0.92, 1.57)	.61
Posttreatment (18 months) vs pretreatment	28	0.72 (−1.20, 2.64)	.47
*HbA1c (%*)			
Posttreatment (6 months) vs pretreatment	60	−0.70 (−1.20, −0.10)	.02*
Posttreatment (12 months) vs pretreatment	46	0.76 (0.09, 1.42)	.03*
Posttreatment (18 months) vs pretreatment	28	0.77 (−0.10, 1.65)	.09

CI = confidence interval, DAAs = direct-acting antiviral agents, HbA1c = hemoglobin A1c, Q1–Q3 = first quartile-third quartile.

* *P*-value <.05 is set as nominally significant.

In our secondary analysis, patients were categorized based on diabetes improvements, evaluated by changes in HbA1c levels and modifications in diabetes medication regimes. Improvement was defined as a reduction in HbA1c of at least 0.5% without alterations in diabetes medications, or a stabilization of HbA1c concurrent with a reduction in diabetes medications. Out of 60 patients, 25 demonstrated improvements in diabetes control post-SVR treatment. In follow-up data ranging from 6 months to 1 year posttreatment, 12 out of 46 patients sustained these improvements, while only 3 out of 28 maintained them at 18 months. Then, we examined potential differences in clinical and demographic variables between patients with and without improvements in diabetes control. No significant differences were observed in gender, insulin prescription, cirrhosis status, age, HCV genotype, total bilirubin, albumin levels, AST, ALT, baseline HCV RNA levels, posttreatment glucose levels, posttreatment HbA1c, hepatorenal biochemical parameters or insulin resistance indicators (as shown in Table 4). Factors associated with diabetes improvement included cirrhosis (16% vs 51.42% in improvement vs non-improvement groups, *P* < .01), BMI (21.30 [20.35, 22.80] vs 25.70 [23.20, 28.60], *P* < .01), pretreatment fasting glucose (8.26 [7.29, 10.58] vs 6.86 [6.13, 8.24], *P* < .05), and pretreatment HbA1c (7.90 [7.00, 9.60] vs 7.00 [6.25, 7.50], *P* < .05) (Table 4).

**Table 4:** Clinical characteristics among patients with improved diabetes after treatment [defined as reduction in HbA1c ≥ 0.5% with no change in diabetic treatment, or no change in HbA1c (<0.5% absolute change) with a reduction in diabetic treatment] versus not improved.

	Diabetes improved	Diabetes not improved	
Chi-square test			
	Percent (%) (N = 25)	Percent (%) (N = 35)	*P*-value
Gender (male)	12 (48%)	18 (51.43%)	.87
Cirrhosis	4 (16%)	18 (51.42%)	<.01*
Prescribed insulin	13 (52%)	11 (31.43%)	.05

Mann–Whitney *U* test			
	Median (Q1–Q3) (N = 25)	Median (Q1–Q3) (N = 35)	*P*-value
Age	65 (55–68)	60 (54–64)	.09
Body mass index (BMI, kg/m^2^)	21.30 (20.35–22.80)	25.70 (23.20–28.60)	<.01*
Total bilirubin (µmol/L)	21.50 (13.00, 38.50)	16.40 (11.95, 25.65)	.62
Albumin (g/L)	38.50 (33.00, 42.70)	37.80 (34.60, 42.70)	.83
AST (U/L)	47.00 (33.00, 115.00)	36.00 (24.50, 61.50)	.25
ALT (U/L)	72.00 (31.00, 181.00)	41,00 (26.00, 84.50)	.16
Pretreatment fasting glucose (mmol/L)	8.26 (7.29, 10.58)	6.86 (6.13, 8.24)	<.01*
Pretreatment HBA1C (%)	7.90 (7.00, 9.60)	7.00 (6.25, 7.50)	<.01*
Pretreatment HCV RNA (10^6^ IU/mL)	2.53 (0.42, 3.93)	2.76 (0.07, 5.45)	.93
Posttreatment glucose (mmol/L)	7.19 (5.77, 9.30)	7.20 (6.62, 8.14)	.88
Posttreatment HbA1c (%)	6.40 (5.90, 7.50)	6.80 (6.30, 7.30)	.52

Mann–Whitney *U* test			
	Median (Q1–Q3) (N = 13)	Median (Q1–Q3) (N = 13)	*P*-value
HOMA-IR	3.01 (1.88–5.14)	3.38 (3.00–4.19)	.55
QUICKI	0.32 (0.30–0.35)	0.32 (0.31–0.32)	.46
Insulin (pmol/L)	49.4 (30.3–79.2)	72.04 (64.5–99.4)	.08

	Median (Q1–Q3) (N = 23)	Median (Q1–Q3) (N = 29)	*P*-value
Total cholesterol (mmol/L)	4.20 (3.44–4.64)	4.22 (3.78–4.96)	.52
Triglycerides (mmol/L)	1.19 (0.87–1.55)	1.25 (0.87–1.76)	.41
HDL cholesterol (mmol/L)	1.15 (0.98–1.37)	1.01 (0.93–1.29)	.48
LDL cholesterol (mmol/L)	2.65 (2.06–3.08)	2.72 (2.40–3.09)	.56
Creatinine (µmol/L)	131.30 (112.55–143.95)	116.70 (100.50–139.00)	.29

ALT = alanine aminotransferase, AST = aspartate aminotransferase, BMI = body mass index, HbA1c = hemoglobin A1c, HCV = hepatitis C virus, HDL = high-density lipoprotein, HOMA-IR = homeostatic model assessment of insulin resistance, IU = international units, LDL = low-density lipoprotein, Q1–Q3 = first quartile-third quartile, QUICKI = Quantitative Insulin Sensitivity Check Index.

* *P*-value <.05 is set as nominally significant.

We also compared other indicators between baseline and 6 months posttreatment, including fasting insulin levels, insulin resistance indicators, hepatorenal biochemical parameters, and the prevalence of renal dysfunction and lipidemia disorder. Only AST (39.00 [24.75–73.25] vs 26.00 [21.00–35.00], *P* < .01) and ALT (54.00 [28.00–97.75] vs 30.00 [17.00–49.00], *P* < .01) showed significant differences before and after treatment (Table S4, Supplemental Digital Content, https://links.lww.com/MD/Q377).

Finally, we conducted stratified analyses of fasting glucose and HbA1c levels at baseline and 6 months posttreatment based on previously identified significant parameters (BMI, cirrhosis status, AST, and ALT). At 6 months posttreatment, only patients with BMI < 24 demonstrated significant reductions in both fasting glucose (*P* = .02) and HbA1c (*P* < .01) from baseline. Similarly, the non-cirrhotic group, but not the cirrhotic group, demonstrated significantly lower fasting glucose (*P* = .02) and HbA1c (*P* < .01) levels at 6 months posttreatment compared to baseline. Both normal (*P* < .01) and abnormal (*P* = .02) AST groups showed lower HbA1c levels posttreatment, with no differences in fasting glucose. Both normal (*P* = .02) and abnormal (*P* = .01) ALT groups showed lower HbA1c levels posttreatment, with no differences in fasting glucose (Table S5, Supplemental Digital Content, https://links.lww.com/MD/Q377).

## 4. Discussion

In this comprehensive analysis, we evaluated the impact of HCV treatment on diabetes management in HCV patients with T2DM. Our findings highlighted the short-term regulatory effects of DAAs on HCV-related diabetes. Moreover, the regulation of CXCL10 protein expression levels during DAAs treatment may synergistically improve HbA1c levels. Notably, our cross-sectional study did not identify a potential association between interferon, ribavirin, and diabetes-related indicators.

HCV infection is an independent risk factor for T2DM, with HCV-induced insulin resistance serving as the key connecting mechanism between the 2 conditions. HCV affects glucose metabolism through 3 main pathways: direct impairment of insulin secretion function in pancreatic β-cells^[27]^; disruption of insulin signaling through promoting TNF-alpha production and enhancing IRS serine phosphorylation, reducing IRS-1 tyrosine phosphorylation,^[28]^ and impairing the PI3 kinase/Akt pathway^[29]^; and induction of a chronic inflammatory state, upregulating pro-inflammatory cytokines while down-regulating anti-inflammatory mediators. Additionally, alterations in key components of insulin signaling pathways, including PI3K, IRS1, MAP3K, AKT, and PTEN, have been observed in HCV-infected cell lines.^[27]^

Currently, there is still a lack of robust clinical studies validating interferon and ribavirin as independent factors in reducing blood glucose or HbA1c levels. On the contrary, interferon-related diabetes may exacerbate T2DM to T1DM, necessitating lifelong insulin therapy.^[30]^ Recent studies suggest that peginterferon alfa and ribavirin might slightly improve T1DM.^[31]^ Moreover, interferon and ribavirin therapy for HCV can cause serious adverse effects, including autoimmune thyroiditis, systemic lupus erythematosus, T1DM, and acute immune rejection in liver transplant recipients.^[32]^ Although some evidence has attempted to demonstrate that interferon and ribavirin might help improve HCV-related diabetes,^[27,33]^ the ultimate conclusions still emphasize successful sustained SVR as the core standard. In patients with SVR failure, glucose abnormalities, progression of liver fibrosis, and even significantly increased risks of hepatocellular carcinoma (HCC) have been noted.^[4]^

DAAs, compared to ribavirin, has been proved superior viral clearance agents, particularly in patients with severe liver disease,^[34]^ highlighting their potential to improve systemic symptoms of HCV. Our retrospective analysis revealed significant short-term improvements in glycemic control (within 6 months), consistent with findings from several prospective and retrospective studies.^[11,28]^ Moreover, mendelian randomization results also suggest that CXCL10 may be a critical target for DAAs in improving diabetes. As a key mediator in immune responses and inflammatory pathways, CXCL10 has been identified as an effective prognostic marker significantly associated with the success of DAA treatment and SVR.^[35]^ The regulation of CXCL10, resulting from DAA treatment, may improve glycemic control through its effects on liver function and metabolic homeostasis.^[36]^ Inhibition of inflammatory factors, including CXCL10 by DAAs, may influence the development of diabetes by reducing insulin resistance and metabolic disorders.

Our study also observed changes in glycemic indicators following DAA treatment, while evaluating changes in other important clinical parameters. AST and ALT levels improved significantly after DAA treatment, consistent with expected outcomes. However, other metabolic parameters, including insulin resistance indicators, renal dysfunction as indicated by elevated creatinine, and lipid metabolism disorders, did not show significant improvement. This suggests that DAA therapy may indirectly influence glucose metabolism through improved liver function, but has minimal effect on insulin secretion or sensitivity. Notably, stratified analysis indicated that short-term HbA1c improved significantly after treatment regardless of whether AST and ALT were normal, suggesting that DAA therapy may have additional mechanisms for regulating glucose metabolism independent of liver enzyme-indicated hepatic inflammatory activity.^[37]^ However, the HbA1c-improving effect of DAAs at 6 months posttreatment disappeared in patients with cirrhosis or abnormal BMI. This indicates that the glycemic benefits of DAAs may be limited in patients with established cirrhosis, overweight/obesity, or concurrent metabolic abnormalities. A large-scale clinical study of 1630 HCV patients with F3 and F4 fibrosis receiving DAA treatment also found that cirrhosis, diabetes, and nonresponse to DAA were the most significant independent risk factors for HCC.^[38]^ A complex synergistic relationship exists among these 3 factors, as cirrhotic patients often present with insulin resistance and glucose metabolism abnormalities, while diabetes may accelerate the progression of cirrhosis to HCC, creating a cycle that DAAs may find difficult to break.

Unfortunately, the long-term sustainability of DAA treatment’s improvement in glycemic and HbA1c has been disappointing. At 12 months posttreatment, average fasting glucose and HbA1c levels had generally returned to pre-DAA treatment levels or even exceeded them. Large-scale multicenter studies have observed that the glucose-improving effects of DAAs or traditional antiviral drugs tend to diminish or become insignificant over time.^[11,12,39]^ We speculated that this may be related to the long-term impact of HCV infection on the metabolic state of patients, the complexity of bodily recovery post-DAA treatment, and the temporal pattern of inflammatory marker regulation. Particularly in patients with baseline insulin resistance, diabetes management improvements required more targeted interventions and multi-pathway regulation.^[40]^ Additionally, our study found that patients with cirrhosis, or those with higher pretreatment HbA1c and fasting glucose levels, were more likely to benefit from short-term glycemic improvements post-DAA treatment. Previous studies similarly indicated that in patients with higher HbA1c, DAAs more significantly improve HCV glucose levels.^[37]^ Guidelines also recommend implementing multifaceted HCV treatment approaches for patients with T2DM.^[41,42]^ Additionally, standard care approaches for metabolic management should be maintained, including diet control, regular exercise, and appropriate use of antidiabetic medications.^[43,44]^ Notably, glucose-lowering and lipid-lowering medications may reduce the risk of liver disease progression and extrahepatic manifestations in HCV diabetic patients. Metformin can significantly reduce the risk of primary liver cancer in patients with cirrhosis or chronic viral hepatitis,^[30,45]^ and statins show similar effects.^[46]^ The importance of statins therapy is particularly emphasized given that DAA treatment may increase LDL levels and cardiovascular risk.^[37]^ However, even after achieving SVR, glycemic control may rebound within 1 to 2.5 years.^[9,11,12]^ On one hand, HCV clearance cannot completely reverse metabolic changes caused by long-term infection, especially in patients with comorbidities such as obesity and hypertension. Previous studies found that insulin resistance was strongly associated with BMI but weakly correlated with SVR,^[30,47]^ consistent with our findings. On the other hand, HCV can cause irreversible pancreatic β-cell damage, and its metabolic alterations further induce hepatic steatosis, associated with fibrosis progression and cardiovascular events.^[48,49]^ The persistence of inflammatory responses after viral clearance emphasizes the necessity of posttreatment metabolic management. For high-risk groups of T2DM, early antiviral treatment combined with healthy lifestyle is key to improving prognosis.^[50]^ In future studies, we plan to establish prediction models based on factors such as cirrhosis, BMI, baseline fasting glucose, and HbA1c, and conduct longitudinal analyses to thoroughly investigate the dynamic patterns of glycemic indicators during treatment and follow-up, guiding individualized treatment strategies.

Our study introduces several novel aspects to the research on HCV treatment effects. First, it is the inaugural investigation that utilizes Mendelian randomization to explore the impact of anti-HCV drug target genes on diabetes. Second, our research firstly utilized NHANES database and explore the association between interferon/ribavirin treatment and diabetes indicators. Third, this is the pioneering study examining the effects of DAAs on HCV-T2D in a Mainland Chinese population. However, our study also has limitations. We lack continuous data on lifestyle and weight changes, limiting our ability to track how these factors affect long-term glucose control. Moreover, all patients received an identical DAA regimen, lacking comparisons across different treatment options. Another limitation is the retrospective nature of our analysis, based on data from a single institution, which may restrict the generalizability of our findings.

## 5. Conclusion

Our study demonstrates that DAAs treatment can improve glycemic control in HCV-infected diabetic patients in the short term (6 months), but this benefit is limited by cirrhosis and BMI, and diminishes after 12 months. Long-term glycemic control of HCV-infected patients with T2DM requires continuous diabetes management and lifestyle modification in addition to antiviral therapy.

## Author contributions

**Conceptualization:** Jian Zhou, Yan Zhao, Guoping Yin, Rui Zhang.

**Data curation:** Yan Zhao, Guoping Yin, Rui Zhang

**Formal analysis:** Rui Zhang.

**Investigation:** Jie Tang, Liyao Zhu.

**Methodology:** Jie Tang, Liyao Zhu, Rui Zhang.

**Project administration:** Guoping Yin, Rui Zhang.

**Resources:** Jie Tang, Guoping Yin.

**Software:** Jie Tang, Guoping Yin.

**Supervision:** Rui Zhang.

**Validation:** Rui Zhang.

**Visualization:** Guoping Yin.

**Writing – original draft:** Jie Tang, Guoping Yin.

**Writing – review & editing:** Guoping Yin, Rui Zhang.

## Supplementary Material




