# Supplementary material for: Direct-acting antiviral treatment impact on glycemic control in HCV-infected T2DM patients: An observational study with Mendelian randomization analysis
Source: Medicine (Baltimore). 2025 Oct 31;104(44):e44319. doi: 10.1097/MD.0000000000044319 (PMC12582676; doi:10.1097/MD.0000000000044319)
Supplement: Supplementary file 1 [file medi-104-e44319-s001.docx]

| **Drug** | **Gene** | **Regulatory approval** | **Interaction score** |
| --- | --- | --- | --- |
| **DAAs** | | | |
| DACLATASVIR | IFNL3 | Not Approved | 2.95 |
| DACLATASVIR | VDR | Not Approved | 0.06 |
| DACLATASVIR | IFNL4 | Not Approved | 3.69 |
| DACLATASVIR | CYP24A1 | Not Approved | 3.69 |
| OMBITASVIR | IFNL3 | Approved | 2.36 |
| OMBITASVIR/PARITAPREVIR/RITONAVIR | IFNL4 | Not Approved | 3.69 |
| OMBITASVIR/PARITAPREVIR/RITONAVIR | IFNL3 | Not Approved | 2.36 |
| OMBITASVIR/PARITAPREVIR/RITONAVIR | IFNL4 | Not Approved | 3.69 |
| OMBITASVIR/PARITAPREVIR/RITONAVIR | IFNL3 | Not Approved | 2.36 |
| PARITAPREVIR | KRAS | Approved | 0.32 |
| PARITAPREVIR | IFNL3 | Approved | 1.18 |
| RITONAVIR | CYP2D6 | Approved | 0.01 |
| RITONAVIR | CYP3A | Approved | 0.11 |
| RITONAVIR | CYP2B6 | Approved | 0.08 |
| RITONAVIR | ERVW-4 | Approved | 0.04 |
| RITONAVIR | UGT1A7 | Approved | 0.36 |
| RITONAVIR | TLR4 | Approved | 0.22 |
| RITONAVIR | NPSR1 | Approved | 0.03 |
| RITONAVIR | VDR | Approved | 0.01 |
| RITONAVIR | CYP3A4 | Approved | 0.02 |
| RITONAVIR | APOC1 | Approved | 4.72 |
| RITONAVIR | ABCC2 | Approved | 0.11 |
| RITONAVIR | ABCB1 | Approved | 0.02 |
| RITONAVIR | KAT2A | Approved | 0.02 |
| RITONAVIR | CYP3A5 | Approved | 0.03 |
| RITONAVIR | APOE | Approved | 0.22 |
| RITONAVIR | APOC3 | Approved | 2.36 |
| RITONAVIR | CYP3A7 | Approved | 0.17 |
| RITONAVIR | CXCL10 | Approved | 0.28 |
| RITONAVIR | CYP3A43 | Approved | 0.17 |
| RITONAVIR | IFNL3 | Approved | 0.09 |
| RITONAVIR | ABCC1 | Approved | 0.08 |
| RITONAVIR | TOMM40 | Approved | 4.72 |
| RITONAVIR | UGT1A3 | Approved | 0.29 |
| RITONAVIR | UGT1A1 | Approved | 0.26 |
| RITONAVIR | CYP2E1 | Approved | 0.16 |
| DASABUVIR | IFNL3 | Approved | 4.72 |
| SOFOSBUVIR | IFNL3 | Approved | 14.16 |
| ELBASVIR | IFNL3 | Approved | 2.36 |
| ELBASVIR/GRAZOPREVIR | IFNL4 | Not Approved | 3.69 |
| ELBASVIR/GRAZOPREVIR | IFNL3 | Not Approved | 2.36 |
| GRAZOPREVIR | IFNL3 | Approved | 1.18 |
| GRAZOPREVIR | KRAS | Approved | 0.32 |
| GRAZOPREVIR | IFNL3 | Approved | 1.18 |
| GRAZOPREVIR | KRAS | Approved | 0.32 |
| LEDIPASVIR | IFNL3 | Approved | 3.54 |
| LEDIPASVIR | IFNL4 | Approved | 3.69 |
| GLECAPREVIR | KRAS | Approved | 0.64 |
| VELPATASVIR | IFNL4 | Approved | 3.69 |
| VELPATASVIR | IFNL3 | Approved | 3.54 |
| **RIBAVIRIN** | | | |
| RIBAVIRIN | IMPDH1 | Approved | 0.23 |
| RIBAVIRIN | CARD16 | Approved | 1.59 |
| RIBAVIRIN | IL6 | Approved | 0.03 |
| RIBAVIRIN | IFIT1 | Approved | 1.59 |
| RIBAVIRIN | VWF | Approved | 0.17 |
| RIBAVIRIN | HLA-B | Approved | 0.13 |
| RIBAVIRIN | BCL2 | Approved | 0.1 |
| RIBAVIRIN | SOCS3 | Approved | 0.4 |
| RIBAVIRIN | SLC28A2 | Approved | 0.4 |
| RIBAVIRIN | MMP1 | Approved | 0.16 |
| RIBAVIRIN | CXCL8 | Approved | 0.02 |
| RIBAVIRIN | VDR | Approved | 0.01 |
| RIBAVIRIN | OAS1 | Approved | 0.53 |
| RIBAVIRIN | IL21R | Approved | 1.06 |
| RIBAVIRIN | KLRK1 | Approved | 0.4 |
| RIBAVIRIN | CYP2R1 | Approved | 0.53 |
| RIBAVIRIN | FTO | Approved | 0.2 |
| RIBAVIRIN | SLC6A4 | Approved | 0.02 |
| RIBAVIRIN | OASL | Approved | 1.59 |
| RIBAVIRIN | CYP27B1 | Approved | 0.32 |
| RIBAVIRIN | DNAAF9 | Approved | 1.06 |
| RIBAVIRIN | CTLA4 | Approved | 0.05 |
| RIBAVIRIN | CST3 | Approved | 1.59 |
| RIBAVIRIN | TUT7 | Approved | 1.59 |
| RIBAVIRIN | TP53 | Approved | 0 |
| RIBAVIRIN | HLA-A | Approved | 0.14 |
| RIBAVIRIN | ANGPT2 | Approved | 0.25 |
| RIBAVIRIN | KRT18 | Approved | 0.8 |
| RIBAVIRIN | ALDH1A1 | Approved | 0 |
| RIBAVIRIN | IL18 | Approved | 0.16 |
| RIBAVIRIN | HLA-C | Approved | 0.09 |
| RIBAVIRIN | VIP | Approved | 0.4 |
| RIBAVIRIN | SLC29A1 | Approved | 0.19 |
| RIBAVIRIN | EGFR | Approved | 0.02 |
| RIBAVIRIN | CASP1 | Approved | 0.12 |
| RIBAVIRIN | MICB | Approved | 0.53 |
| RIBAVIRIN | RORC | Approved | 0.02 |
| RIBAVIRIN | EGFR | Approved | 0.02 |
| RIBAVIRIN | CASP1 | Approved | 0.12 |
| RIBAVIRIN | MICB | Approved | 0.53 |
| RIBAVIRIN | RORC | Approved | 0.02 |
| **INTERFERON** | | | |
| INTERFERON ALFACON-1 | IFNAR1 | Approved | 4.66 |
| INTERFERON ALFACON-1 | IFNAR2 | Approved | 6.94 |
| INTERFERON ALFA-2B | IFNAR2 | Approved | 0.99 |
| INTERFERON ALFA-2B | TP53 | Approved | 0.02 |
| INTERFERON ALFA-2B | IFNA2 | Approved | 0.42 |
| INTERFERON ALFA-2B | TGFB1 | Approved | 0.15 |
| INTERFERON ALFA-2B | IFNAR1 | Approved | 0.67 |
| INTERFERON ALFA-2B | FTO | Approved | 0.53 |
| INTERFERON ALFA-2B | ITPA | Approved | 2.11 |
| INTERFERON ALFA-2B | EIF2AK2 | Approved | 2.11 |
| INTERFERON ALFA-2B | IFNG | Approved | 0.45 |
| INTERFERON ALFA-2B | IFNL4 | Approved | 0.79 |
| INTERFERON ALFA-2B | CSF2 | Approved | 0.11 |
| INTERFERON ALFA-2B | IL6 | Approved | 0.17 |
| INTERFERON ALFA-2B | BAX | Approved | 0.13 |
| INTERFERON ALFA-2B | IL18 | Approved | 0.42 |
| INTERFERON ALFA-N3 | IFNAR1 | Approved | 3.1 |
| INTERFERON ALFA-N3 | IFNAR2 | Approved | 3.47 |
| INTERFERON ALFA-N3 | IFNA2 | Approved | 1.97 |
| INTERFERON ALFA-N1 | IFNAR1 | Not Approved | 1.55 |
| INTERFERON ALFA-N1 | IFNAR2 | Not Approved | 2.6 |
| INTERFERON ALFA-N1 | IFNL4 | Not Approved | 1.84 |
| INTERFERON ALFA-N1 | IFNL3 | Not Approved | 1.18 |

**Supplementary Table 1. Target genes information of Interferon, ribavirin, and DDAs from the DGIdb database.**

**ABCB1 = ATP-binding cassette subfamily B member 1; ABCC = ATP-binding cassette subfamily C; APOC = Apolipoprotein C; APOE = Apolipoprotein E; CYP = Cytochrome P450; DAAs = Direct-acting antiviral agents; ERVW-4 = Endogenous retrovirus group W member 4; IFNAR = Interferon alpha and beta receptor; IFNL3/4 = Interferon lambda 3/4; KRAS = Kirsten rat sarcoma viral oncogene homolog; KAT2A = Lysine acetyltransferase 2A; NPSR1 = Neuropeptide S receptor 1; TLR4 = Toll-like receptor 4; TOMM40 = Translocase of outer mitochondrial membrane 40; UGT = UDP glucuronosyltransferase; VDR = Vitamin D receptor.**

| **Characteristic** | **All NHANES participants with information of interferon or ribavirin treatment (n=37)** | | | |
| --- | --- | --- | --- | --- |
|  | **N** | | **%** | |
| **Age** | | | | |
| **60- years** | 26 | | 70.27% | |
| **60+ years** | 11 | | 29.73% | |
| **Gender** | | | | |
| **Male** | 26 | | 70.27% | |
| **Female** | 11 | | 29.73% | |
| **Race/ethnicity** | | | | |
| **Mexican American** | 4 | | 10.81% | |
| **Other Hispanic** | 2 | | 5.41% | |
| **Non-Hispanic white** | 21 | | 56.76% | |
| **Non-Hispanic Black** | 9 | | 24.32% | |
| **Other/multiracial** | 1 | | 2.70% | |
| **BMI** | | | | |
| **Underweight (<18.5)** | 1 | | 2.70% | |
| **Normal (18.5 to <24)** | 4 | | 10.81% | |
| **Overweight (24 to <30)** | 18 | | 48.65% | |
| **Obese (30 or greater)** | 14 | | 37.84% | |
| **Alcohol intake** | | | | |
| **<1 drinks/month** | 16 | | 43.24% | |
| **1 to <5 drinks/month** | 15 | | 40.54% | |
| **<10 drinks/month** | 6 | | 16.22% | |
| **Smoking status** | | | | |
| **<100 cigarettes/life** | 31 | | 83.78% | |
| **＞100 cigarettes/life** | 6 | | 16.22% | |
| **Educational attainment** | | | | |
| **Less than 9th grade** | 2 | | 5.41% | |
| **9–11th grade (12th grade**  **with no diploma)** | 4 | | 10.81% | |
| **High school graduate/**  **GED** | 174 | | 470.27% | |
| **Some college or AA** | 7 | | 18.92% | |
| **College graduate or**  **above** | 10 | | 27.03% | |
| **Treated with medication such as interferon and ribavirin** | | | | |
| **Yes** | 15 | | 90.00% | |
| **No** | 22 | | 3.33% | |
|  | **Mean** | **SD** | **Median** | **Q1-Q3** |
| **Age (years)** | 52.33 | 9.87 | 51.00 | 46.00-60.25 |
| **Fasting glucose (mg/dL)** | 111.56 | 36.42 | 102.50 | 90.00-121.00 |
| **HbA1c (%)** | 5.77 | 0.99 | 5.50 | 5.28-6.00 |
| **Insulin (μIU/mL)** | 18.28 | 14.06 | 11.59 | 8.64-24.29 |

**Supplementary Table 3. Characteristics of enrolled HCV participants with self-reported information of interferon or ribavirin treatment from NHANES dataset.**

**AA = Associate degree; BMI = Body mass index; GED = General Educational Development; HbA1c = Hemoglobin A1c; NHANES = National Health and Nutrition Examination Survey; Q1-Q3 = First quartile-Third quartile; SD = Standard deviation**

|  | **Pre-treatment** | | **Post-treatment** | |  |
| --- | --- | --- | --- | --- | --- |
| **Wilcoxon signed-rank test** | | | | | |
|  | **Median (Q1-Q3) (N = 26)** | | **Median (Q1-Q3) (N = 26)** | | **p-value** |
| **HOMA-IR** | 3.35 | (2.00-4.76) | 3.42 | (1.87-5.06) | 0.91 |
| **QUICKI** | 0.32 | (0.30-0.34) | 0.32 | (0.30-0.35) | 0.64 |
| **Insulin (pmol/L)** | 69.60 | (42.84-87.98) | 79.70 | (41.95-99.70) | 0.29 |
|  | **Median (Q1-Q3) (N = 52)** | | **Median (Q1-Q3) (N = 52)** | | **p-value** |
| **Total Cholesterol (mmol/L)** | 4.21 | (3.71-4.85) | 4.42 | (3.72-4.75) | 0.79 |
| **Triglycerides (mmol/L)** | 1.24 | (0.87-1.69) | 1.215 | (0.90-1.45) | 0.79 |
| **HDL Cholesterol (mmol/L)** | 1.10 | (0.94-1.35) | 1.15 | (0.99-1.33) | 0.99 |
| **LDL Cholesterol (mmol/L)** | 2.70 | (2.27-3.10) | 3.02 | (2.31-3.543) | 0.54 |
| **Creatinine (µmol/L)** | 124.80 | (103.13-140.05) | 128.50 | (103.98-136.13) | 0.63 |
| **Total bilirubin (μmol/L)** | 17.90 | (12.10-27.68) | 16.60 | (12.00-25.50) | 0.09 |
| **Albumin (g/L)** | 38.50 | (33.00-42.93) | 41.50 | (39.00-44.40) | 0.19 |
| **AST (U/L)** | 39.00 | (24.75-73.25) | 26.00 | (21.00-35.00) | **＜0.01#** |
| **ALT (U/L)** | 54.00 | (28.00-97.75) | 30.00 | (17.00-49.00) | **＜0.01#** |
| **Chi-square test** | | | | | |
|  | **Percent (%) (N = 52)** | | **Percent (%) (N = 52)** | | **p-value** |
| **Renal Dysfunction** | 24 (46.15%) | | 26 (50.00%) | | 0.70 |
| **Lipidemia Disorder** | 38 (73.08%) | | 41 (78.85%) | | 0.49 |

**Supplementary Table 4. Wilcoxon signed-rank test of Insulin resistance metrics, lipid profile and hepatorenal biochemical parameters before and after intervals of DAAs treatment, and Chi-square analysis of Renal dysfunction and Lipidemia Disorder before and after DAAs treatment.**

**#p-value < 0.05 is set as nominally significant**

**ALT = Alanine aminotransferase; AST = Aspartate aminotransferase; HDL = High-density lipoprotein; HOMA-IR = Homeostatic Model Assessment of Insulin Resistance; LDL = Low-density lipoprotein; Q1-Q3 = First quartile-Third quartile; QUICKI = Quantitative Insulin Sensitivity Check Index**

| **Wilcoxon signed-rank test** | | | | |
| --- | --- | --- | --- | --- |
|  | | **Median (Q1-Q3) (Pre-treatment)** | **Median (Q1-Q3) (Post-treatment)** | **p-value** |
| **BMI < 24** | **fasting Glucose (mmol/L)** | 7.92 (6.50-10.14) | 7.12 (5.70-8.46) | **0.02#** |
| **BMI < 24** | **HbA1c (%)** | 7.40 (6.50-8.05) | 6.80 (5.90-7.25) | **＜0.01#** |
| **BMI > 24** | **fasting Glucose (mmol/L)** | 7.47 (5.99-8.67) | 7.47 (6.83-8.14) | 0.58 |
| **BMI > 24** | **HbA1c (%)** | 7.20 (6.30-7.70) | 6.60 (6.30-7.50) | 0.39 |
| **Cirrhosis** | **fasting Glucose (mmol/L)** | 7.77 (5.95-9.92) | 8.02 (6.84-11.56) | 0.40 |
| **Cirrhosis** | **HbA1c (%)** | 7.45 (6.93-9.50) | 6.85 (6.45-8.40) | 0.35 |
| **Non-Cirrhosis** | **fasting Glucose (mmol/L)** | 7.60 (6.50-9.00) | 7.19 (6.33-8.06) | **0.02#** |
| **Non-Cirrhosis** | **HbA1c (%)** | 7.20 (6.30-7.80) | 6.70 (6.10-7.30) | **＜0.01#** |
| **AST (Normal)** | **fasting Glucose (mmol/L)** | 7.55 (6.54-9.55) | 7.20 (6.81-8.42) | 0.22 |
| **AST (Normal)** | **HbA1c (%)** | 7.20 (6.30-8.10) | 6.60 (6.10-7.40) | **＜0.01#** |
| **AST (Abnormal)** | **fasting Glucose (mmol/L)** | 7.33 (6.45-9.65) | 7.34 (6.77-8.57) | 0.38 |
| **AST (Abnormal)** | **HbA1c (%)** | 7.20 (6.30-7.95) | 6.60 (6.10-7.43) | **0.02#** |
| **ALT (Normal)** | **fasting Glucose (mmol/L)** | 7.60 (5.82-8.57) | 7.13 (5.90-8.14) | 0.07 |
| **ALT (Normal)** | **HbA1c (%)** | 7.40 (6.45-7.70) | 6.70 (6.00-7.41) | **0.02#** |
| **ALT (Abnormal)** | **fasting Glucose (mmol/L)** | 7.99 (5.92-8.78) | 7.15 (5.96-8.08) | 0.77 |
| **ALT (Abnormal)** | **HbA1c (%)** | 7.45 (6.38-7.93) | 6.80 (5.90-7.35) | **0.01#** |

**Supplementary Table 5. Wilcoxon signed-rank test analysis of fasting glucose and HbA1c levels before and after 6-month DAAs treatment intervals, stratified by BMI, cirrhosis status, and liver function parameters (AST&ALT).**

**#p-value < 0.05 is set as nominally significant.**

**ALT = Alanine aminotransferase; AST = Aspartate aminotransferase; BMI = Body mass index; HbA1c = Hemoglobin A1c; Q1-Q3 = First quartile-Third quartile.**
